# Comparative Study between an Immediate Loading Protocol Using the Digital Workflow and a Conventional Protocol for Dental Implant Treatment: A Randomized Clinical Trial

**DOI:** 10.3390/jcm8050622

**Published:** 2019-05-07

**Authors:** Piyanut Rattanapanich, Weerapan Aunmeungtong, Pisaisit Chaijareenont, Pathawee Khongkhunthian

**Affiliations:** 1 Center of Excellence for Dental Implantology, Faculty of Dentistry, Chiang Mai University, Chiang Mai 50200, Thailand; piyanasss@hotmail.com (P.R.); weedentphd@outlook.com (W.A.); 2 Department of Prosthodontic, Faculty of Dentistry, Chiang Mai University, Chiang Mai 50200, Thailand; yodent@hotmail.com

**Keywords:** immediate loading, digital implant workflow, dental implant treatment, CAD/CAM, patient satisfaction

## Abstract

*Background*: The purposes of this randomized clinical trial study was to compare the immediate loading of dental implants while employing digital workflow and conventional implants in terms of the success rate, marginal bone level, and patient satisfaction. *Methods*: Fifty patients who had edentulous area on the mandibular premolar or molar area were included in the study. Twenty-five patients were assigned to immediate loading implant treatment using the digital technique and 25 patients were assigned to conventional loading implant treatment. In the first group, the patients were received digital impression (Cerec Omnicam, Dentsply Sirona^®^, York, PA, USA), designed, producing zirconia crown, and inserted on the same surgery day. The second group, after a healing period of three months, was received analog impression following conventional impression for the zirconia crown. Clinical outcome and radiographic bone level were evaluated after three, six, and 12 months. Patient satisfaction was measured at 12 months after inserting the implant. *Results*: There was no implants and protheses failure in both groups. The mean resonance frequency analysis values at the day of surgery were 78.26 ± 4.09 in immediate loading using the digital group (ILD) and 73.74 ± 5.14 in the conventional loading group (CL), respectively. Insertion torque values at the day of surgery were 36.60 ± 12.64 in ILD and 38.8 ± 12.19 CL, respectively. The marginal bone level in CL at three, six, and 12 months were 0.14 ± 0.28 mm, 0.18 ± 0.30 mm, and 0.17 ± 0.29 mm, respectively, while in ILD at three, six, and 12 months were 0.18 ± 0.33 mm and 0.16 ± 0.27 mm and 0.15 ± 0.31, respectively. There was no statistically significant difference between the two groups. Only one question in patient satisfaction’s questionnaire was “Now, can your dental implant and crown be used well?” had been significantly different in favor to the conventional group. *Conclusion*: Within the limitation of this study, it may be concluded that, after one-year follow up, there were no statistically significant differences between the immediate loading of dental implants employed from the digital workflow and conventional implant treatment technique in the success rate and marginal bone level. In patient satisfaction, there was only statistic significant difference in question related to implant prosthetic function in favor of the CL group, whereas the question concerning speaking, cleansing, price, and expectation displayed no difference.

## 1. Introduction

Osseointegrated titanium dental implants have been successfully used to completely and partially restore edentulous areas [1,2,3,4,5].

The development of materials, surface characterisitcs, shapes, and designs of dental implants continues [6,7,8]. In general, the long-term survival rates of dental implants are very high. Therefore, the use of a single implant for a single tooth replacement has been increasing, especially in the posterior region. Dental implants have been widely used to support fixed dental prostheses, and the dental profession is confident in regards to their use.

Pjetursson et al. conducted a systematic review in 2004 to follow the survival rate of implants for implant-supported fixed partial dentures. At five years, the survival rate was 95.4%, and at 10 years, 92.8%. Later, they reported a systematic review in 2012, including 32 studies, showing the survival rate of implant-supported fixed dental prostheses at five- and 10-year follow-up, as 95.6% and 93.1%, respectively. Excluding machined-surface implants, the survival rates increased to 97.2% and 95.4%, at five and 10 years, respectively [9].

Weber et al. and Esposito et al. have proposed implant loading protocols [10,11]. Conventional loading: in the traditional scenario, the prosthesis is attached to the implant following a period of at least two months after the placement of the implant. Immediate loading: the attachment of the prosthesis to the implant occurs within a week of the placement of the implant. Early loading: the attachment process occurs at any time from one week to two months after placing the implant.

Linkow introduced the immediate loading of implants in 1963. The first self-tapping endosseous root-form implants received loading with overdentures or provisional fixed bridges [12].

The immediate loading of implants permits the delivery of fixed prostheses to be accomplished on the same day as implant placement. Such implant therapy has received significant gratification and acknowledgement from patients, as it decreases the amount of time that is required for the treatment, averting a second- stage operation, as well as providing immediate comfort due to the fact that it is not necessary to have a temporary removable prosthesis while the healing phase is being conducted.

Moraschini et al. performed a meta-analysis of studies investigating the marginal bone level and survival rates of implants while using both conventional and immediate approaches to loading for single implants that are located in the posterior mandible. No statistically significant differences were found between groups in the case of either survival or bone loss [13].

Esposito et al. conducted further meta-analysis in order to compare the conventional and immediate loading one-year after the event. The patient completion rate was 100% and each of the two groups recorded only a single failure throughout the course of these studies. No statistically significant differences were found between the two groups for either prosthesis failure or for implants [14].

For the processes of implant dentistry, digital techniques are increasingly popular in the diagnosis and planning of the treatment, as well as during the actual procedures themselves. Furthermore, digital approaches can support the design stage of crowns and they can guide the production process using milling machines, which offer the necessary precision to allow for the crown to be immediately inserted on completion of surgery. These factors account for the increasingly widespread use of these techniques.

In the area of restorative dentistry, Duret et al. were the first to use techniques from computer-aided design or manufacturing (CAD/CAM) as long ago as 1971. CAD/CAM technology is now more widely used in dentistry, especially in implant restorations or for implant abutment fabrication. The advantage of using this kind of technology is that it requires less time and work to complete the process than conventional therapy, thus providing convenience and cost savings [14,15].

Joda and Bragger examined the usage of ready-made abutments in order to compare these with CAD/CAM abutments involving monolithic crowns that are designed and produced with a milling machine for a single-tooth implant. Their work revealed that it is not necessary to grind the proximal or occlusal aspects of the crowns or abutments in the cases where a digital system is used in the design. This process can now be completed in just two steps: recording the digital impression and the insertion of the crown [16]. 

Since it is apparent that advantages exist when using immediate loading along with digital technology, it can be argued that dentists would be well-advised to develop these approaches for use in their own practice when working with dental implants. However, the number of studies that involve immediate loading combined with digital technology is limited and no researchers have yet examined immediate loading in the context of permanent zirconia crowns. Accordingly, it was decided to conduct this study.

The objective of this randomized clinical trial study was to compare the immediate loading of dental implants employing the digital workflow, and conventional implants in terms of success rate, marginal bone level, and patient satisfaction. The research null hypothesis was that there is no significant difference between an immediate loading protocol while using digital workflow and a conventional protocol for dental implant treatment.

## 2. Experimental Section

This study was designed as randomized clinical trial. The Human Experimentation Committee, Office of Research Ethics, Faculty of Dentistry, Chiang Mai University provided ethical approval for this study (number 11/2560). Registration as a Thai clinical trial was completed under the registration number TCTR20170919001.

### 2.1. Patients Selection

The target population was males or females over 20 years of age who had a space in the mandibular premolar or molar regions, including occluded teeth. The sample size was calculated as that *n* = 20 for each group using G*Power (version 3.1.9.4, Heinrich-Heine-Universität Düsseldorf, Düsseldorf, Germany) as the statistical power analysis. For preliminary patient inclusion, a sample size of 50 patients, which were randomly selected from the patients visiting to either immediate loading using digital group (ILD) and conventional loading group (CL), was determined to be acceptable for reaching the level of required statistical power. All of the patients provided their consent having been informed of the details of the study. This was obtained before the inclusion screening process. The selection of participants was made at random, with patients being chosen from those attending the Centre of Excellence in Dental Implantology at the Faculty of Dentistry, Chiang Mai University. Table 1 shows the inclusion and exclusion criteria.

**Figure 1:**
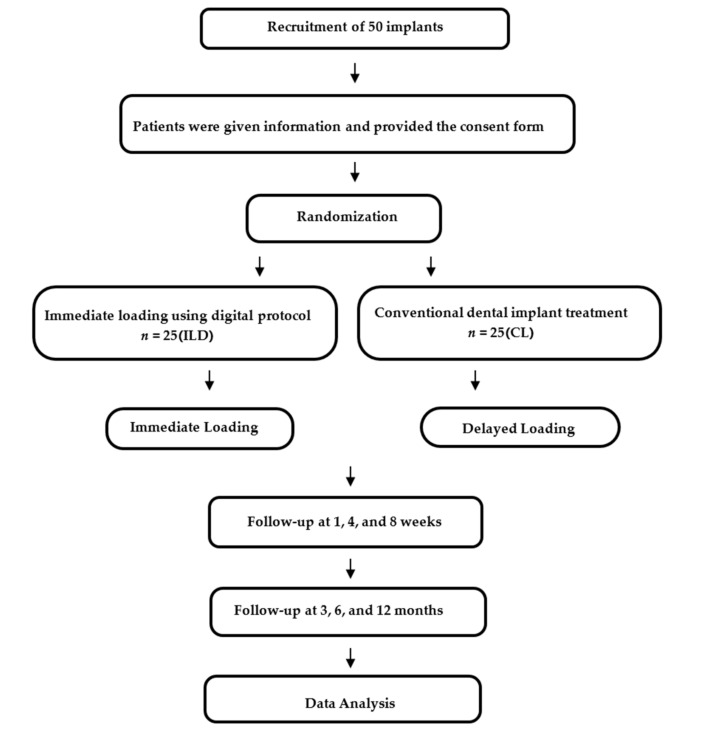
Flowchart for the research.

The study also investigated further factors using radiographic evaluations, considerations of mobility and complications, and finally, the level of patient satisfaction when such surgery was performed in the region of the mandibular premolars or molars. In this study, two categories were identified for the subdivision of the population, as shown in Figure 1. Following the placement of the dental implants, follow-up visits were organized after one, four, eight, and 12 weeks, and then after six and 12 months. The soft tissue was examined, any implant complications were noted, the implant stability was checked, and any prosthodontic problems were addressed. Periapical radiography was performed during the three, six, and 12-month check-ups, so that any changes in the bone levels near the implants could be compared. Patient satisfaction after 12 months was evaluated through the use of a questionnaire while using a visual analog scale (VAS), with a range of 0–100.

### 2.2. Surgical Procedures

Surgical procedures were performed under local anesthesia with 3.6 ml of 4% articaine with 1:100,000 epinephrine. A surgical stent was inserted to indicate the implant position since the approach was flapless. A drill size was chosen in line with the instructions of the manufacturer of the dental implant.

The implant (NDI, PW Plus, Thailand), with a diameter of 4.2 mm and 10 mm in length, was tightened with a torque wrench into the prepared site. The insertion torque was required to be greater than 30 Ncm. The insertion torque was embedded at the bone level. The resonance frequency analysis (RFA) values and insertion torque values were recorded on the day of surgery through a periapical film for the purpose of checking upon the dental implant. The same surgeon performed all of the operations.

### 2.3. Prosthetic Procedures

**Figure 2:**
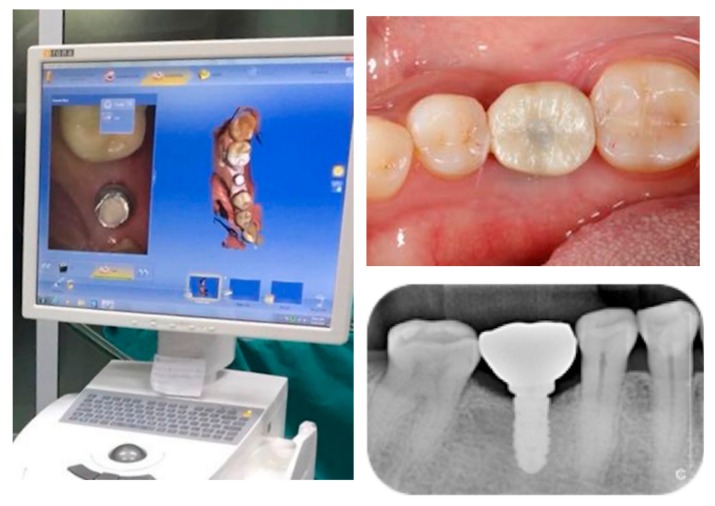
Shows intraoral scanning with CEREC AC Connect with CEREC Omnicam (Dentsply Sirona^®^, York, PA, USA).

**Figure 3:**
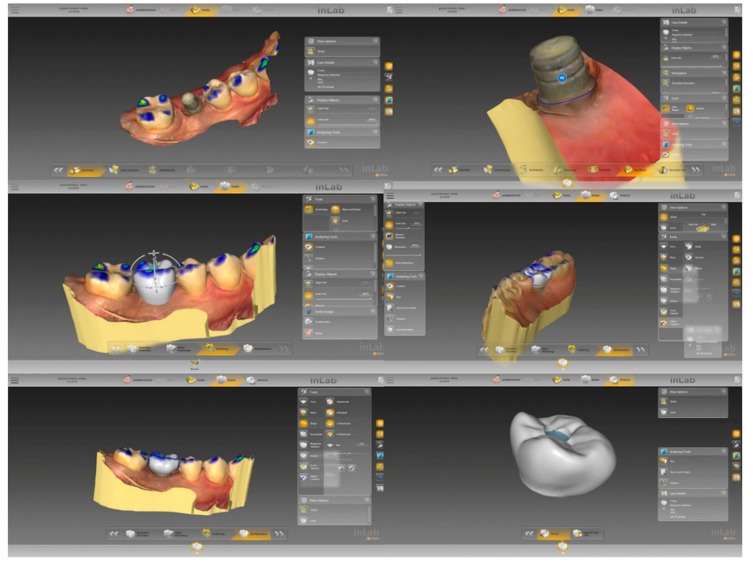
Screwed-retained crown was designed from Inlab SW programme and sent to milling machine (MCX5, Dentsply Sirona^®^, York, PA, USA).

For the sample group undergoing ILD group, the impressions were recorded while using an intraoral scanner and the data were employed in the computer-assisted design (CAD) stage in order to design the crown, which was then fabricated using computer-assisted manufacturing (CAM) in a milling machine, as shown in Figure 2 and Figure 3. The crown was designed to be held in place while using a screw. The crown was then inserted, once the location had been determined, following an evaluation of the occlusal vertical dimension and the occlusal surface. The implant was fitted on the titanium abutment. The researcher sought to prevent the cases of eccentric occlusion by making corrections to occlusion errors, so that a stable bite could be established in centric occlusion. The sample group CL group followed the conventional process. These crowns were then implanted using abutment screws, which were tightened on two occasions, ten minutes apart, using a torque of 30 Ncm.

### 2.4. Parameters for Measurement

Assessment of the Success Rate

The Pisa Consensus Conference 2007 criteria were employed to evaluate implant success at 12 months [17].

Success (optimal health outcome):

- functionality without pain or tenderness;
- zero mobility;
- radiographic bone loss resulting from surgery not exceeding 2 mm; and,
- absence of exudate history.

Radiographic Evaluation

**Figure 4:**
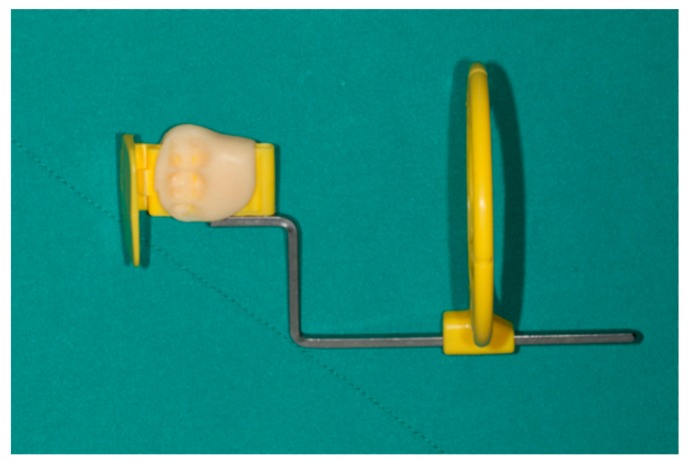
The customized occlusal bite jig was fabricated attach to film holder in each patient.

The first periapical radiograph was obtained as soon as the implant is installed together with the insertion of the prosthesis in the immediate loading group and the implant is installed in the control group, being used as a baseline for further comparisons at three, six, and 12 months. To evaluate the marginal bone level, a customized occlusal bite jig was constructed for each patient while using the parallel radiographic technique, which could be used on each visit with exactly the same position and angulation to allow for a comparison to be drawn over time (Figure 4). Two examiners were calibrated for marginal bone level measurement.

Patient Satisfaction

A questionnaire was used after 12 months to determine the extent to which the patients were satisfied with the treatment and outcomes [18]. Eleven questions were included, as shown in Table 2.

### 2.5. Statistical Analysis

The null hypothesis in this study was that no significant difference exists at the twelve-month follow-up between ILD and CL, in terms of success rate, marginal bone level, and patients’ satisfaction. The success rate was considered as the primary outcome. The sample size chosen was calculated to provide a normal distribution; 25 patients were needed in order to determine a 95% confidence interval for the group. The data analysis was performed using SPSS 17.0 software (SPSS Inc., Chicago, IL, USA). The data distribution was tested using the Shapiro–Wilk test for normality. The principal data item concerned the success rates for each of the treatment groups. The implant stability quotients (ISQ) values and the insertion torque for the two groups at the time of insertion were recorded. An evaluation of the radiographic bone level was also conducted. The reliability of the measurements of each examiner was evaluated twice using the intraclass correlation coefficient (ICC). Repeated ANOVA was used to compare the changes taking place within the same group over time at specified intervals, and independent sample t-testing was used to make comparisons between the groups (*p* ≤ 0.05). The patient satisfaction questionnaire was then analyzed in order to provide the VAS score

## 3. Results

Thirty-eight female patients and 12 male patients were included in this study. The mean age was 50.38 years (SD = 14.21). The entire sample group completed the treatment and participated until the end of the study. No implant failure, disintegration, or prosthetic failure were observed. Patient demographics are shown in Table 3.

The mean ISQ values in the ILD and CL groups were 78.26 ± 4.09 and 73.74 ± 5.14, respectively. The insertion torque values on the insertion day were 36.60 ± 12.64 and 38.8 ± 12.19 for the ILD and CL groups, respectively (Table 4).

**Figure 5:**
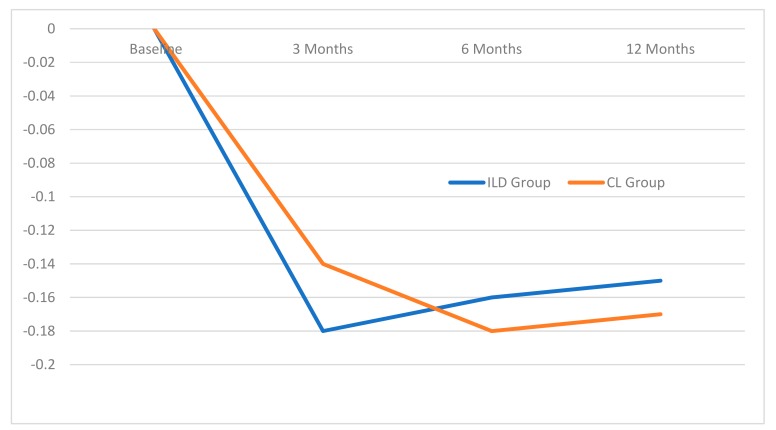
Marginal bone levels at the 3, 6 and 12-month intervals.

Mean marginal bone level in the CL group at the three, six, and 12-month intervals were 0.14 ± 0.28 mm, 0.18 ± 0.30 mm, and 0.17 ± 0.29 mm, respectively. In the ILD group, the bone loss values were 0.18 ± 0.33 mm, 0.16 ± 0.27, and 0.15 ± 0.31, respectively (Figure 5). There were no statistical differences between both of the groups and each follow-up time.

Table 5 shows the VAS scores regarding the patient satisfaction. The first question was statistical difference and greater satisfaction in the CL group (*p* = 0.004), and the others presented no statistic significant differences.

## 4. Discussion

There have been a number of studies that advocate the use of immediate loading, citing the lowered treatment time and the positive psychological effect upon patients, as well as the undiminished success rate when compared to conventional loading. In the mandibular area, the immediate loading success rates were in the range of 79–100%, while for restorations in both jaws, the figure was 96–100% [19].

Chrcanovic et al. conducted a systematic review along with a meta-analysis of eleven related research studies. These studies indicated no significant influence upon the implant failure rate for the immediate functional and non-functional loading [20].

Guidetti et al. provided a report regarding immediate functional loading implants in a study that involved twelve patients; the study achieved a 12-month survival rate of 83.3% [21].

However, the present study used randomized clinical trials in order to make comparisons of success rate, prosthesis failure, and complications. There were 100% success rates in both the ILD and CL group. Neither prosthetic failures nor biological complications were found.

The marginal bone levels showed no significant differences, and there were no reports of values lower than 1 mm at the follow-up point at three, six, and 12 months. Engelhardt et al. conducted a systematic review examining the marginal bone level, finding that the mean differences in weight between immediate and conventional loading was just 0.02 mm after one year (*p* > 0.05), rising to 0.08 after two years (*p* > 0.05), then −0.10 mm after three years (*p* > 0.05), and −0.3 mm after five years (*p* < 0.05). The differences observed were not clinically relevant in terms of radiographic bone changes in level after five years [22].

Giacomel et al. conducted randomized control trials in order to compare the changes in the marginal bone level for three implant types: immediate loaded implants, delayed loaded non-submerged implants, and delayed loaded submerged implants. No statistically significant differences were reported between these types at the follow-up after nine months [23].

Hence, the digital workflow, together with the immediate loading protocol, has no influence on the marginal bone level. The marginal bone levels in our study are between 0.1 to 0.2 mm, which may come from physiologic bone remodeling. However, the long-term observation is still required.

It is clear that the role played by digital technology in the dental field is very useful, especially in allowing much faster restorations for patients. Joda et al. conducted a randomized control trial in order to make a comparison of the treatment time efficiency that involved lithium disilicate crowns made using the CAD/CAM process without modeling, and porcelain that was fused to zirconium dioxide crowns that were constructed through the conventional digital approach. It was also found that the chair and laboratory time were significantly reduced [24]. From our study, we used the all of the zirconia restoration with a required milling time of about 40 minutes and sintering time of about 120 minutes. The patients had to wait for about three hours for inserting a crown in ILD group. There are also some materials that need less production time than all zirconia crowns, however the strength of the materials has to be considered for their use in the molar area.

Zembic et al. conducted randomized control trials to investigate survival rates, which also examined the technical and biological complications that are associated with zirconia and titanium abutment and the follow-up appointment five years after the insertion of crowns. The findings indicated a 100% survival rate and, while chipping was observed in veneering ceramics where metal-ceramics were employed, no significant differences were reported. From our study, there was no found prosthetic failure. All of the patients had the crowns completely inserted within three hours after the surgery [25].

This study took advantage of both digital technology and immediate loading to complete the implant treatment for study participants. The process of designing and preparing permanent zirconia crowns and subsequently inserting the implants through immediate loading was completed within a single day. The shortened treatment time may be one of the important factors in facilitating the patient making the decision to have the implant treatment in the future.

The results from this study examined the level of satisfaction of dental implant therapy; there was statistical difference favouring the CL group in first question, which implied the use of implant prosthesis. This may result from the reluctance to use the implant prosthesis in the IL group immediately after surgery, when compared to the CL group. Nevertheless, the question regarding speaking, cleansing, price, and expectation displayed no statistical differences. Whilst, when comparing to other studies, Pjetursson et al. reported a prospective cohort study of dental implant with a 10-year follow-up regarding function and chewing. The comfort of dental implant showed high patient’s satisfaction. VAS score was 94 ± 13. The phonetics and esthetics were highly satisfying. Trouble of cleansing was not reported [18]. Similarly, Lee et al. used modified Oral Health Impact Profile (OHIP) in single-tooth implant treatment and they found that patients were satisfied with pronouncing, but they had trouble with discomfort. However, it was not assured that the discomfort was related to the prostheses or surgery [26].

Within the limitations of this study, neither immediate loading using the digital group nor conventional group had no statistic significant difference in success rate. However, the clinical study of using digital workflow together with implant treatment are necessary in evaluating the long-term survival rate of implant and its prostheses.

## 5. Conclusions

From our randomized clinical trial, it may be concluded that, after one-year follow-up, there were no statistical significant differences between the immediate loading of dental implants employing digital workflow and conventional implant treatment techniques in the success rates and marginal bone levels. In patient satisfaction, there were only statistically significant differences in questions related to the implant prosthetic function favouring the CL group, whereas the question concerning speaking, cleansing, price, and expectation presented no differences.

## Figures and Tables

**Table 1:** Criteria used for inclusion or exclusion in the study.

Inclusion Criteria	Exclusion Criteria
**General Criteria**	
1. Missing permanent teeth between the premolar and mandibular molar, which have been extracted for more than 4 months	1. Patients suffering systemic diseases that might have reduced their chances of completing the procedure causing them to drop out of the study
2. Permanent teeth present with stable occlusion.	2. Patients who had frequently used antibiotics or steroids
3. Patients must have been in sufficiently good health to undergo surgical procedures	3. Patients with disorders involving blood platelets, or showing unusual or inadequate levels of erythrocytes or leucocytes
4. Patients should have shown no previous signs of psychosis	4. Patients with HIV
5. Patients must have been non-smokers or have smoked fewer than 10 cigarettes daily during the previous five years	5. Patients who were in poor health and could undergo surgery, who had psychosis, or disorders which could lead to bleeding with an uncontrollable risk level in excess of ASA III
6. Patients should have good levels of dental hygiene	6. Patients who smoked more than 10 cigarettes daily or are alcoholics
7. Patients with positive emotions towards dental implants and with a good understanding of the implant procedures	7. Patients who had undergone treatment involving radiation in the area of the jaw or neck, or had received chemotherapy
8. Patients who could undergo treatment and attend no fewer than 6 to 7 follow-up meetings	8. Patients who were pregnant
9. Patients who can offer their informed consent in writing	9. Patients who expressed negative feelings towards the notion of dental implants
	10. Patients who failed to maintain adequate dental hygiene levels
	11. Patients who could attend for treatment or could attend follow-up meetings
	12. Patients who did not give informed consent in writing
**Dental Implant Criteria**	
1. There was no oral soft or hard tissue pathosis and the patient exhibited excellent dental health	
2. Patients who had normal soft tissue in the oral cavity and their keratinized mucosa had a width of at least 4 mm	
3. The bone surrounding the implant should have exhibited a labio-lingual and bucco-lingual width of at least 6 mm with a height of at least 12 mm; therefore, surgery would not have been necessary to strengthen the bone prior to insertion of the implant	

**Table 2:** Questionnaire for evaluating patient satisfaction.

1.	Does your dental implant and crown allow good functional use?
2.	Which are better for chewing: the dental implant, or natural teeth?
3.	Are you able to speak normally?
4.	Are you satisfied with the way you look?
5.	Are you able to clean the implant easily?
6.	Is it easier to clean the dental implant than natural teeth?
7.	Is it faster to clean the dental implant, or the natural teeth?
8.	Has the dental implant treatment performed as expected?
9.	Compared to the opportunity to wear dentures, would you prefer to undergo the dental implant treatment?
10.	Would you encourage friends or family to have the dental implant treatment?
11.	Do you believe that the dental implant price is suitable?

**Table 3:** Patient Demographics.

Patient Demographic	Immediate Loading Using Digital Workflow	Conventional Loading
Male/Female	4/21	8/17
Mean age	49.16 ± 11.07	51.60 ± 16.44
First molar/second molar	25/0	24/1

**Table 4:** Mean and SD for ISQ and insertion torque at the time of surgery.

Implant Stability Measurements	ILD Group (*n* = 25)	CL Group (*n* = 25)
	Mean ± SD	Mean ± SD
ISQ	78.26 ± 4.09	73.74 ± 5.14
Insertion torque	36.60 ± 12.64	38.80 ± 12.19

ISQ = implant stability quotients.

**Table 5:** Mean and standard deviation of visual analog scale (VAS) score from patient satisfaction.

Questionnaires	Group	Mean ± SD	*t*-test *p* Value
Question 1	CL	94.76 ± 9.45	0.004
	ILD	86.34 ± 10.34	
Question 2	CL	63.15 ± 24.20	0.756
	ILD	65.12 ± 20.55	
Question 3	CL	85.54 ± 22.44	0.832
	ILD	86.79 ± 18.93	
Question 4	CL	87.07 ± 17.61	0.946
	ILD	87.39 ± 14.49	
Question 5	CL	76.25 ± 18.26	0.923
	ILD	76.76 ± 19.47	
Question 6	CL	64.23 ± 26.78	0.975
	ILD	64.01 ± 21.59	
Question 7	CL	77.13 ± 23.20	0.146
	ILD	68.41 ± 18.25	
Question 8	CL	95.05 ± 8.56	0.161
	ILD	91.97 ± 10.32	
Question 9	CL	94.32 ± 8.61	0.363
	ILD	92.02 ± 8.87	
Question 10	CL	94.99 ± 9.05	0.450
	ILD	93.11 ± 9.39	
Question 11	CL	75.07 ± 20.98	0.271
	ILD	68.51 ± 20.73	
